# Prodromal Sleep Disturbances and Polysomnographic Findings in Patients With Creutzfeldt–Jakob Disease

**DOI:** 10.1002/brb3.70971

**Published:** 2025-12-17

**Authors:** Ezgi Demirel, Gul Yalcin‐Cakmakli, Ezgi Yetim, Serap Saygi, Fadime Irsel Tezer

**Affiliations:** ^1^ Department of Neurology Hacettepe University Faculty of Medicine Ankara Turkey

**Keywords:** apnea, Creutzfeldt–Jakob disease, neurodegenerative disorders, polysomnography, sleep problems

## Abstract

**Purpose:**

Prion diseases are fatal neurodegenerative disorders of the central nervous system. Sporadic Creutzfeldt–Jakob disease (sCJD) is the most encountered form of the disease. The mean survival time is less than a year after diagnosis. Sleep problems are common in CJD. However, only a few studies revealed sleep pathology of CJD.

**Method:**

Between 2013 and 2023, patients diagnosed with probable CJD who had video electroencephalography (EEG) and polysomnography (PSG) studies were evaluated. Detailed examination of the first and last EEGs (if any) was based on background activity, slow wave paroxysms, epileptiform discharges, and additional findings. Macro‐ and microstructure of sleep and sleep disturbances were evaluated with PSG studies.

**Results:**

Seven patients (two females, five males) at the mean age of 60 years were analyzed. Gait disturbances, difficulty in speech, and visual problems were common in addition to progressive cognitive impairment. Neurological examination revealed ataxia, dysarthria, myoclonus and/or aphasia, and loss of vision. Typical 1 Hz generalized periodic discharges and slow wave activity on the background were encountered in the advanced stage of the disease. Sleep‐related problems were reported by three patients (42.8%); however, when we questioned carefully, we realized all patients had sleep disturbances such as simple or complex movements during sleep, sleep vocalizations, insomnia, and daytime sleepiness simultaneously with the main symptom. During PSG recording, sleep‐related apnea and insomnia were frequently reported. Interestingly, one patient had an initial de novo finding of Cheyne–Stokes apnea.

**Conclusion:**

Sleep problems such as insomnia or apnea could be the first sign of CJD. It should be carefully questioned of every patient and caregiver. PSG may help to specify prognostic and clinical differences.

## Graphical abstract

This study investigates the sleep‐related manifestations of Creutzfeldt–Jakob disease (CJD) by analyzing electroencephalography (EEG) and polysomnography (PSG) findings in a cohort of seven patients diagnosed between 2013 and 2023. Although only three patients initially reported sleep‐related complaints, comprehensive assessments revealed that all participants exhibited sleep disturbances—ranging from parasomnia and vocalizations to insomnia and excessive daytime sleepiness—co‐occurring with core neurological symptoms. EEG analyses showed progressive deterioration of background activity, with the appearance of slow waves and 1 Hz periodic discharges in advanced stages. PSG recordings revealed frequent sleep‐related breathing abnormalities, disrupted microstructure of sleep, and rapid eye movement (REM) parasomnias, with one case demonstrating Cheyne–Stokes respiration as an early, de novo finding. These results highlight the importance of detailed sleep assessment in suspected CJD cases, suggesting that REM parasomnias and apneas may serve as early clinical indicators, and that PSG could aid in both diagnostic and prognostic stratification.

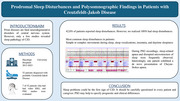

## 1 Introduction

Prion diseases are a group of fatal, rapidly progressive neurodegenerative disorders, characterized by the accumulation of misfolded prion protein (PrPSc). Prion diseases are categorized as sporadic, genetic, or acquired. Approximately 85% of cases are sporadic. Sporadic Creutzfeldt–Jakob disease (sCJD) is the most common form of the disease, caused by a somatic mutation in *PRNP* or the spontaneous conversion of PrPC to PrPSc (Zerr and Parchi 2018).

Age at onset of CJD shows a peak between 55 and 75 years with a median of 67 years (Nafe et al. 2023). Median survival time is nearly 5–6 months. Diagnosis is based on clinical features and confirmation of PrPSc presence. Progressive cognitive impairment, myoclonus, visual or cerebellar problems, pyramidal or extrapyramidal features, and akinetic mutism are common clinical features.

The use of cerebrospinal fluid biomarker 14‐3‐3, unique electroencephalography (EEG) patterns showing periodic sharp‐wave complexes, and observable MRI features such as restricted diffusion seen on diffusion‐weighted imaging (DWI) in neocortical areas and the basal ganglia region are supportive of the diagnosis with variable sensitivity and specificities (Altuna et al. 2022). Aside from brain biopsy, the Real‐Time Quaking‐Induced Conversion (RT‐QuIC), a highly sensitive (80%–96%) and ultraspecific (99%–100%) in vitro PrPSc amplification assay, is the sole antemortem diagnostic biomarker specific for the disease, directly identifying the pathological prion protein (PrPSc) and since 2018 has been incorporated in the current diagnostic criteria (Altuna et al. 2022).

Sleep disturbances are a common and significant feature of neurodegenerative diseases, including conditions such as insomnia, disrupted sleep‐wake cycles, excessive daytime sleepiness, obstructive and central sleep apnea, REM sleep behavior disorder, and restless legs syndrome (Iranzo 2016). In prion diseases, sleep problems such as reduced sleep spindles and slow‐wave sleep, along with vivid dreaming during REM sleep, are well known in fatal familial insomnia (Montagna et al. 2003). However, only few studies focus on sleep disturbances in early CJD patients. In a case series, it is shown that early‐stage E200K familial CJD patients exhibit distinct pathological sleep patterns such as spindle loss, reduced REM and slow‐wave sleep, abnormal respiratory events, and the absence of muscle atonia (Givaty et al. 2016). In a retrospective study, it is highlighted that sleep dysfunction was present in nearly all the patients, although a small number had premorbid sleep diagnoses (Kang et al. 2016a). These findings suggest that abnormal sleep patterns may serve as early biomarkers of the disease spectrum. The purpose of this research was to record and describe sleep disruptions in our group of patients diagnosed with CJD, and to explore any connections between these disturbances and the severity of their other cognitive and motor symptoms.

## 2 Materials and Methods

This study included seven patients diagnosed with CJD in our tertiary center between February 2013 and February 2024. All the patients have met the diagnostic criteria of probable CJD (Hermann et al. 2021) and undergone video EEG and polysomnography (PSG) recordings. Demographic characteristics, clinical features, duration of symptoms, cerebrospinal fluid (CSF) studies, features of neuroimaging and electrophysiological studies are noted. Detailed information about sleep disorders such as insomnia, hypersomnia, parasomnia, sleep‐related movement disorders, snoring, sleep apnea was obtained from family members or sleep partners.

PSG was performed for 1 or 2 days to all patients. EEG signals were captured using a 32‐channel EEG system (Grass‐Telefactor, XLTEK), with scalp electrodes positioned in accordance with the standard 10–20 system. Three chin electromyography (EMG) leads were additionally placed to monitor submental muscle tone, while right and left electrooculogram leads were utilized to track eye movements. EMG leads were also positioned on the right and left tibialis anterior muscles to evaluate leg movements. Routine placement of electrocardiogram, thoracoabdominal excursions, nasal airflow, thermistor, pulse oximetry, and body position belts were conducted.

The macrostructure of sleep and related events were assessed following the guidelines of the American Academy of Sleep Medicine (AASM). Total sleep time (TST), time taken to fall asleep (sleep onset latency), time taken to enter rapid eye movement (REM) sleep (REM latency), the proportion of different non‐rapid eye movement (NREM) sleep stages (N1, N2, N3), and REM sleep, as well as sleep efficiency (SE) and sleep maintenance efficiency were noted. Parameters such as apnea types, apnea–hypopnea index (AHI), mean O_2_ saturation, and percentage of O_2_ saturation below 88% were utilized to assess sleep‐related breathing disorders. Periodic limb movement index, loss of atony in REM, REM sleep behavior disorder (RBD), and parasomnia were also evaluated. All the parameters of sleep were analyzed by an experienced clinical neurophysiologist and sleep specialist.

This study adhered to the principles outlined in the Declaration of Helsinki. Approval was obtained from the Ethics Committee of our university.

## 3 Results

### 3.1 Demographic and Clinical Features

Patients’ characteristics and clinical findings are summarized in Table 1. Mean age of the patients at symptom onset was 60.2 years (ranged between 42 and 73). Female to male ratio was 2:5. Mean time between diagnosis and symptom onset was 3.4 (ranged between 1 and 7) months. Family history was unremarkable in six patients. Patient 1 whom we detected the E200K mutation in his diagnostic evaluation had a significant family history of CJD in his four cousins. Since they lived in other countries, we couldn't obtain sufficient information about them.

**TABLE 1:** Demographic and clinical features of the patients.

	Patients (*n = *7)
Age, years, mean ± SD	60.3 ± 11. 4
Gender, male, *n* (%)	5 (71.4%)
Duration of symptoms, months, mean ± SD	3.4 ± 2.5
Symptoms at onset	
Balance and gait impairment	71.4%
Speech disturbances	71.4%
Visual problems	57.1%
Forgetfulness	28.5%
Neurological examination findings	
Ataxia	57.1%
Dysartria	42.8%
Aphasia	42.8%
Vision loss	42.8%
Myoclonus	42.8%
Pyramidal findings	14.2%
Parkinsonism	14.2%
Sleep problems in medical history	
Simple or complex movements during sleep	42.8%
Insomnia and daytime sleepiness	28.5%
Behavioral changes during sleep	28.5%
Vocalizations	14.2%

*Note*: Values are presented as mean ± standard deviation (SD) or number (*n*) with percentage (%).

Abbreviations: SD: standard deviation, *n*: number; %: percentage.

Most prominent symptoms at disease onset were gait disturbances (71.4%), difficulty in speech (71.4%), and visual problems (57.1%). Although two patients had memory deterioration in the beginning, all subjects developed progressive cognitive impairment in the full‐blown disease. Typical features in neurological examination were ataxia (57.1%), dysarthria (42.8%), aphasia (42.8%), loss of vision (42.8%), myoclonus (42.8%), and less commonly pyramidal findings (14.2%) and Parkinsonism (14.2%).

Sleep‐related problems were reported by three patients (42.8%) among initial symptoms; however, persistent and more careful questioning revealed that all patients had sleep disturbances such as simple or complex movements during sleep, sleep vocalizations, insomnia, and daytime sleepiness. Nevertheless, since the symptoms were queried retrospectively and sleep disorders are not given enough attention by patients, we prefer to describe them as early or concomitant disturbances rather than strictly prodromal.

### 3.2 Diagnostic Investigations

CSF examination, MRI, and EEG assessments were obtained for all the patients. CSF protein was mildly elevated in 71.4% of patients. Mean CSF protein was 54.0 ± 23.6 mg/dL (Table 2). Antibody panel for limbic (LGI1, CASPR2, NMDAR, AMPA‐R, GABA‐B R, DPPX) and paraneoplastic (Anti‐Hu [ANNA‐1], Anti‐Yo [PCA‐1], Anti‐Ri [ANNA‐2], Anti‐Ma2/Ta, Anti‐Amphiphysin, Anti‐CV2.1 [CRMP5], Anti‐Recoverin, Anti‐SOX1 [AGNA], Anti‐GAD65) encephalitis was investigated during the diagnostic process and found to be negative in all the patients. CSF tau protein was elevated with a median of 7602 [1126–10116] pg/mL (Table 2). MRI examinations showed diffusion restriction in at least one region of the brain in most, but not all, of the patients (71.4%). The cerebral cortex (57.1%) and basal ganglia (42.8%) were the most prominent regions (Table 2).

**TABLE 2:** Summary of paraclinical results including laboratory and MRI findings used in diagnostic evaluation.

	Patients (*n = *7)
CSF protein level (mg/dL), mean ± SD	54.0 ± 23.6
CSF total tau protein level (pg/mL), (*n = *5), median [IQR]	7602 [1126–10116]
Limbic panel, any positive result, *n/N* (%)	0/7 (0%)
Paraneoplastic panel, any positive result, *n/N* (%)	0/7 (0%)
Diffusion restriction in MRI (%)	71.4 %
Cerebral cortex (%)	57.1%
Basal ganglia (%)	42.8%
Thalamus (%)	14.2%

*Note*: Values are presented as mean ± standard deviation (SD), median with interquartile range [IQR], or number (*n*) with percentage (%), as appropriate.

Abbreviations: CSF: cerebrospinal fluid; IQR: interquartile range; MRI: magnetic resonance imaging; *n*: number; *N*: total number; %: percentage; SD: standard deviation.

### 3.3 Electroencephalography and Polysomnography Findings

Baseline and follow‐up EEG findings were summarized in Table 3. Background slowing was the most common EEG abnormality. Only 28.5% of patients had normal 9–10 Hz posterior dominant alpha activity in the initial EEG. In addition to the background slowing, non‐epileptiform interictal abnormalities such as intermittent focal or generalized slowing were also prominent in 42.8% of patients in the initial EEG. One patient was diagnosed with non‐convulsive status epilepticus (NCSE) treated with levetiracetam, lacosamide, and topiramate after loading dose of diazepam. A median of 0.8 months later, six patients had follow‐up EEGs, which showed an increase in the severity of background slowing. None of the patients had a normal posterior dominant rhythm. Even so, typical 1 Hz generalized periodic discharges (GPDs) were seen in only half of the patients.

**TABLE 3:** Baseline and follow‐up electroencephalographic characteristics of the patients.

Parameters	Baseline (*n = *7)	Follow‐up (*n = *6)
Background rhythm (Hz), median [IQR]	7.5 [6.0–9.1]	5.5 [4.2–6.4]
≥ 8 Hz*, n* (%)	3 (42. 8%)	0 (0.0%)
4–7 Hz*, n* (%)	4 (57.1%)	5 (83.3%)
< 4 Hz*, n* (%)	0 (0%)	1 (16.7%)
Normal PDR (9–10 Hz), *n* (%)	2 (28.5%)	0 (0%)
Non‐epileptiform interictal abnormalities (paroxysmal focal or generalized slowing), *n* (%)	3 (42.8%)	0 (0%)
Epileptiform discharges (positive sharp waves), *n* (%)	3 (42.8%)	4 (66.7%)
LPDs, *n* (%)	1 (14.2%)	0 (0%)
GPDs, *n* (%)	1 (14.2%)	4 (66.7%)
NCSE, *n* (%)	1 (14.2%)	—
Time between first and last EEG (months), median [IQR]	—	0.8 [0.5–1.8]

*Note*: Values are presented as mean ± standard deviation (SD), median with interquartile range [IQR], or number (*n*) with percentage (%), as appropriate.

Abbreviations: GPD: generalized periodic discharges; IQR: interquartile range; LPD: lateralized periodic discharges; NCSE: non‐convulsive status epilepticus; PDR: posterior dominant rhythm.

All the patients had a PSG assessment for sleep problems, which are summarized in Table 1. Total sleep time, sleep and REM latency, sleep efficiency and continuity, macro‐ and microstructure of sleep, and sleep arousal index were indicated in Table 4. Total sleep time varied between 110 and 437 min with a mean of 343.2 min. Sleep efficiency and sleep continuity were diminished dramatically in most of the patients (71.4%). Disruption of microstructure of sleep (loss of sleep spindles, rare K complexes, rapid transitions between stages) was observed in all the patients. In 28.5% of patients, the sleep microstructure was so severely disrupted that a distinct sleep pattern could not be obtained.

**TABLE 4:** Polysomnographic findings related to macro‐ and microstructure analysis, sleep‐related breathing, and motor abnormalities of sleep.

Parameters	Value
Total sleep time (min), mean ± SD	343.2 ± 74.0 (*n = *5)
Sleep latency (min), mean ± SD	56.2 ± 52.1 (*n = *5)
Sleep efficiency (%), mean ± SD	58.8 ± 30.3 (*n = *5)
Sleep continuity (%), mean ± SD	61.6 ± 28.2 (*n = *5)
REM latency (min), mean ± SD	205.6 ± 106.9 (*n = *5)
N1 (%), mean ± SD	55.2 ± 32.4 (*n = *5)
N2 (%), mean ± SD	37.6 ± 31.8 (*n = *5)
N3 (%), median [IQR]	1.0 [0.0–1.0] (*n = *5)
REM (%), mean ± SD	5.8 ± 1.8 (*n = *5)
REM latency (min), mean ± SD	205.6 ± 106.9 (*n = *5)
Sleep arousal index (number of arousals per hour), mean ± SD	18.5 ± 13.7 (*n = *5)
Sleep apnea, *n*/*N* (%)	7/7 (100%)
Central apnea, *n*/*N* (%)	3/7 (42, 8%)
Central and obstructive apnea *n*/*N* (%)	4/7 (57.1%)
Cheyne–Stokes pattern, *n*/*N* (%)	1/7 (14.2%)
Apnea–hypopnea index (number of apneas–hypopneas per hour), mean ± SD	34.7 ± 28.1 (*n = *6)
Mean O_2_ saturation (%), mean ± SD	91.4 ± 2.7 (*n = *5)
% time O_2_ < 88%, mean ± SD	19.8 ± 20.0 (*n = *6)
Periodic limb movement index (number of periodic limb movements per hour), median [IQR]	0.0 [0.0–44.8] (*n = *7)
Loss of REM atonia, *n*/*N* (%)	3/7 (42.8%)
RBD, *n*/*N* (%)	1/7 (14.2%)
NREM parasomnia, *n*/*N* (%)	0/7 (0%)

*Note*: Values are presented as mean ± standard deviation (SD), median with interquartile range [IQR], or number (*n*) with percentage (%), as appropriate. Two patients did not yield a distinct sleep pattern on PSG; therefore, stage‐based parameters were not computed for these cases and were excluded from quantitative summaries.

Abbreviations: CJD: Creutzfeldt–Jakob disease; IQR: interquartile range; NREM: non‐rapid eye movement; O2 sat: oxygen saturation; RBD: REM sleep behavior disorder; REM: rapid eye movement; SD: standard deviation.

Stages of sleep could be informative in five patients. All had a dramatically prolonged N1 phase. In a patient, there was a noticeable change in the macrostructure of sleep in the follow‐up recording. On the first recording day, N3 stage was dramatically prolonged, whereas on the second one, it was almost absent. REM stage was strikingly shortened in both (Table 4).

**FIGURE 1:**
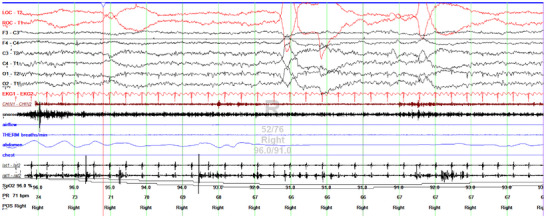
Polysomnography example from a 52‐year‐old man with familial CJD (E200K homozygous)—presenting with unsteadiness, dysarthria, and ophthalmoplegia, with insomnia, increased daytime sleepiness, and nightmares emerging three months later. PSG demonstrated severely fragmented sleep with rapid REM–N1/arousal transitions, marked central and obstructive apneas, and loss of REM atonia consistent with RBD. This representative 30‐s epoch of REM sleep demonstrates loss of REM atonia. The EEG and EOG channels display typical REM sleep features, including low‐amplitude mixed‐frequency EEG activity and rapid eye movements (LOC–ROC). However, there is prominent phasic electromyographic activity in both the chin (mentalis muscle) and right anterior tibialis muscles, consistent with REM sleep behavior disorder. Respiratory signals (nasal airflow, thermistor, abdomen, chest) and oxygen saturation (SpO_2_) are provided for context. CJD: Creutzfeldt–Jakob disease; EEG: electroencephalogram; EMG: electromyogram; EOG: electro‐oculogram; LAT–RAT: left/right anterior tibialis; LOC: left outer canthus; POS: position; PR: pulse rate; PSG: polysomnography; RBD: REM sleep behavior disorder; REM: rapid eye movement; ROC: right outer canthus; SpO_2_: peripheral oxygen saturation. THERM: thermistor.

**FIGURE 2:**
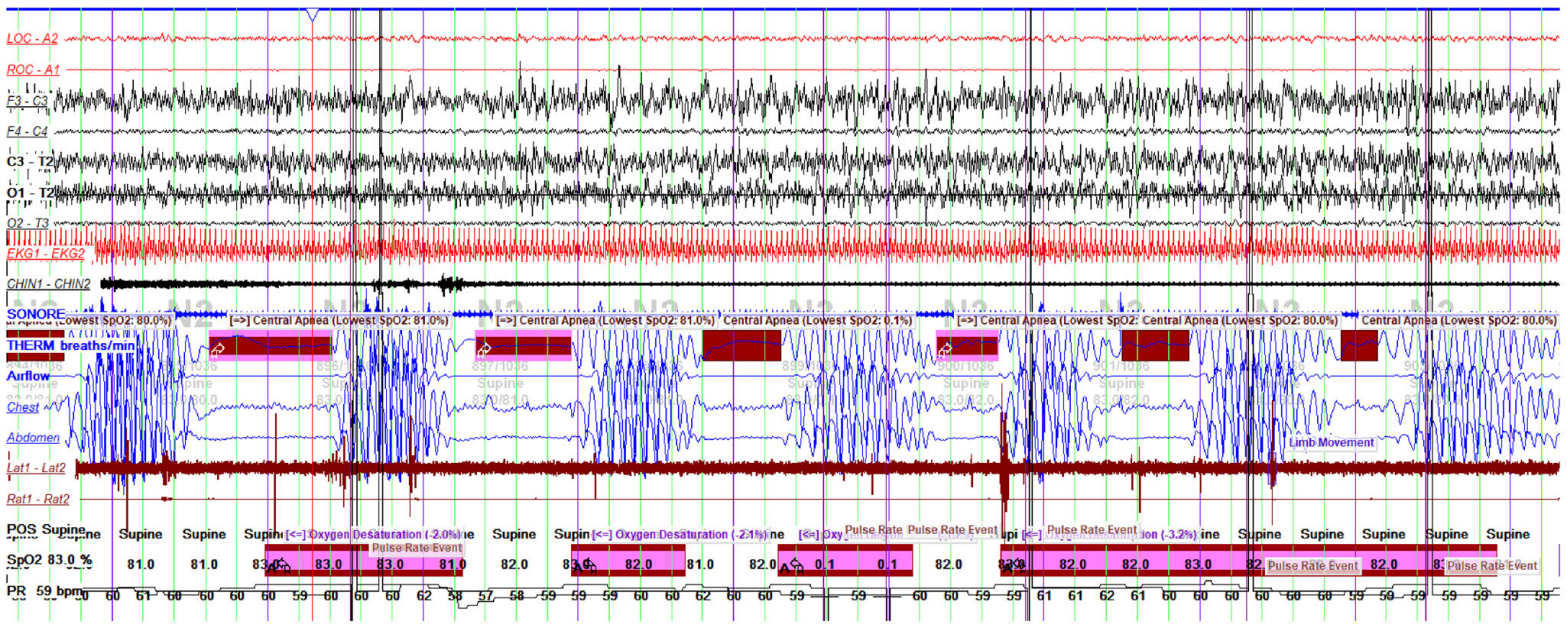
A demonstrative 4‐min 30‐s polysomnography epoch showing central sleep apneas with a Cheyne–Stokes respiration pattern. The patient was a 67‐year‐old man with dysarthria, insomnia, forgetfulness, and Parkinsonism on neurological examination. His MRI was unremarkable other than mild cerebral atrophy. PSG‐video EEG monitoring was performed at the initial phase of the disease due to the suspicion of autoimmune encephalitis. This diagnosis was later excluded during follow‐up. PSG revealed insufficient nocturnal sleep and increased daytime sleep. Microstructure of sleep was also disturbed with loss of K complexes and spindles, although vertex sharp waves were preserved. REM sleep was occasionally disrupted by N1 intrusions or arousals, accompanied by loss of atonia. In this epoch the tracing shows cyclic crescendo–decrescendo fluctuations in respiratory effort and airflow, accompanied by repeated central apneas (highlighted in red boxes). Periodic desaturations in arterial oxygen saturation (SpO_2_) and accompanying pulse rate fluctuations were evident. The patient remained in a supine position throughout the recording. He was eventually diagnosed with Creutzfeldt–Jakob disease (CJD), supported by elevated CSF total tau level (1126 pg/mL), the presence of protein 14‐3‐3 in CSF, and a positive RT‐QuIC test for PrPSc. LAT–RAT: left/right anterior tibialis; LOC: left outer canthus; ROC: right outer canthus; POS: position; PR: pulse rate; THERM: thermistor.

None of these patients had a history of sleep apnea before hospitalization but all of them had sleep apnea, although the mean oxygen saturation was above 88 in most of the patients. In all patients, apnea was either central or mixed (Table 4). One patient had Cheyne–Stokes pattern throughout his sleep (Figure 1, 2). AHI was elevated in 71.4% of patients (Table 4). Also, 42.8% of patients had an increased periodic limb movement index. Loss of atony in REM was prominent in three participants (42.8%), one of them was diagnosed with REM sleep behavior disorder. Non‐REM parasomnia was not seen in any of the patients (Table 4).

## 4 Discussion

In this single‐center retrospective study, we analyzed sleep features of our CJD cohort who had a PSG study between 2013 and 2023. The cohort consisted of six probable sporadic and one genetic CJD patients. Despite only three out of seven patients reported sleep problems, we determined by a detailed history that all patients had sleep problems. Simple or complex movements during sleep and insomnia/daytime sleepiness were the most common findings, followed by behavioral changes and vocalizations. In PSG recordings, the microstructure of sleep was disrupted in all patients. K complexes and sleep spindles were almost absent. Even though the mean oxygen saturation was above 85%, all the patients had sleep apnea either central or mixed. But central apnea was more frequent. We didn't observe NREM parasomnia in any of the patients but three of them had loss of atonia during REM sleep and one of them had clinical RBD.

Current diagnostic criteria for probable CJD include rapidly progressive cognitive impairment and two of the neurological signs (visual or cerebellar disturbance, pyramidal or extrapyramidal signs, myoclonus, akinetic mutism), plus at least one positive biomarker (14‐3‐3 protein in CSF or MRI or EEG) or progressive neuropsychiatric syndrome and positive RT‐QulC (Hermann et al. 2021). In the case of sCJD, sleep problems and PSG findings are not recognized as a diagnostic criterion yet and there are only few studies regarding sleep pathophysiology. However, sleep problems like insomnia, daytime sleepiness, apnea, or RBD may be a part of prodromal phase of CJD (El Sammak et al. 2022; Ogawa et al. 2011). Meissner et al. (2004) reported almost 50% of patients with definite CJD had sleep problems. Kang et al. (2016b) showed that in later stages, the prevalence of sleep problems such as hypersomnia, insomnia, restless legs or nocturnal limb movements, and parasomnia reached up to 89%. A prospective study of seven patients with definite sCJD also clarified severe EEG abnormalities during sleep with loss of sleep spindles, decreased sleep efficiency, and the absence of REM sleep, suggesting a prominent overlap with FFI (Landolt et al. 2006). It is not surprising to see sleep problems in these patients developing encephalopathy at the later stages of the disease. In contrast to that, our patients had initial sleep problems at the first stages of the disease. Central apneas and especially Cheyne–Stokes pattern in one patient are noticeable before the whole clinical picture of CJD.

The detailed PSG findings were not frequently reported in patients with CJD. Earlier sleep EEG and PSG studies in case reports demonstrated that the physiological states of sleep were replaced by periodic complexes alternating with theta‐delta activity (Calleja et al. 1985; Terzano et al. 1995). Similar to our findings, disorganized sleep architecture with loss of K complexes, spindles, and the absence of REM stages were noted (Donnet et al. 1992). Apnea, especially central apnea, was remarked (Kazukawa et al. 1987). We also recorded central apnea in our patients and Cheyne–Stokes pattern was an interesting finding at the initial period of the disease. Parasomnia, however, was rarely reported in CJD (Puligheddu et al. 2017; Hongo et al. 2019). We had one patient with RBD but loss of atonia in other three patients might be a marker of the development of REM parasomnia rather than NREM parasomnia.

Sporadic familial insomnia (the MM2T subtype sCJD based on genotype of the *PRNP* gene) is the most frequent type of prion disease with severe sleep problems such as decreased sleep time and disorganized sleep structure (Parchi et al. 1999). The microstructural elements of sleep, such as sleep spindles and K complexes, are generated by the thalamus (De Gennaro and Ferrara 2003; Halász 2016). So, it is not surprising that in thalamic variants of CJD, sleep structure is highly disrupted and insomnia may be the first sign of the disease (Sun et al. 2021). Sleep problems can sometimes be so severe that they can mimic anti‐IgLON5 disease (Hongo et al. 2019). Similarly, two of our patients were clinically suspected to have anti‐IgLON5 disease.

Moreover, there are genetic CJD cases with E200K mutation whose clinical and PSG characteristics were similar to those described in FFI (Taratuto et al. 2002; Chen et al. 2021). E200K mutation, the glutamic acid (E) to lysine (K) substitution at codon 200, is the most common *PRNP* mutation worldwide with varying penetrance between 59.5% and 100% (Ladogana and Kovacs 2018). Peripheral neuropathy (Neufeld et al. 1992) and thalamic hyperintensities (Kovacs et al. 2009) are distinctive features similar to one of our patient. Sleep problems are common in patients with E200K mutation, so it is also named as thalamic‐insomnia phenotype (Ye et al. 2022; Cohen et al. 2015). Additionally, central apnea or mixed apnea were detected at much higher rates compared to normal population (Cohen et al. 2015). Our patient also had severe obstructive and central apnea together with loss of atony in REM (Figure 1).

Apnea has an impact not only on sleep EEG but also on wakefulness EEG. In patients with obstructive sleep apnea (OSA), EEG slowing during REM sleep is evident in the frontal, central, and parietal areas, whereas during wakefulness, the slowing extends across all cortical regions (Morisson et al. 1998). This slow frequency EEG activity in patients with OSA can be reversed by continuous positive airway pressure (CPAP) (Wang et al. 2021). Similarly, it has been shown that alpha power is increased and the delta/alpha ratio is decreased in REM sleep by CPAP treatment in patients with central sleep apnea syndrome (Zhang et al. 2021). EEG changes of apnea have been linked to apnea‐related sleep fragmentation, repeated episodes of low oxygen, and frequent cortical arousals (Zhou et al. 2020). Moreover, it has also been revealed that the degree of change in hypercapnia is the only significant predictor for slow frequency EEG activity (Wang et al. 2016).

While decreased sleep efficiency, greater sleep fragmentation, and disturbances in sleep microstructure are common polysomnographic findings across various dementias, central apnea is not typically observed in other types such as Alzheimer's disease or frontotemporal dementia (Sani et al. 2019; Zhang et al. 2022). Central apnea is typically related to heart failure, brain stem dysfunction, and chemoreflex instability (Roberts et al. 2022). It is presumed to be caused by brainstem dysfunction in patients with CJD, although it has not been fully clarified (Miyagawa et al. 2018). Apnea is frequently observed in sCJD, particularly in those who also present with other signs of brainstem involvement such as dysarthria and dysphagia (Iwasaki et al. 2006). Central sleep apnea and Cheyne–Stokes respiration in CJD reflect underlying brainstem dysfunction; however, in multisystem atrophy—a related neurodegenerative disorder with prominent brainstem involvement—the respiratory phenotype is more often characterized by stridor, OSA, and hypoventilation unlike CJD. Gascon‐Bayarri (2010) reported PrP deposition in brainstem involving respiratory nuclei in a patient with CJD who had the Cheyne–Stokes pattern both awake and during sleep with central apneas in the initial phase of the disease. Cheyne–Stokes pattern was a prominent and early finding in one of our cases who had dysarthria and sleep problems as initial symptoms. He was hospitalized for aspiration pneumonia secondary to dysphagia in his later stage.

Preclinical studies also support all our clinical findings related to sleep in patients with CJD, considering the role of PrP in sleep physiology. It has been shown that in PrP knocked‐out mice increased degree of slow wave activity and sleep fragmentation was observed (Tobler et al. 1997). Circadian activity patterns and rhythms also change in mice devoid of PrP (Tobler et al. 1996). Mice expressing mutant PrP showed sleep abnormalities such as decreased REM sleep in both phases of the light–dark cycle and reduced NREM sleep during the dark phase (Dossena et al. 2008).

This study had several limitations. Although the disease is rare, the sample size was relatively small, which may restrict the statistical power and generalizability of the findings. Furthermore, the heterogeneity of our cohort, encompassing both sporadic and genetic CJD cases, may have introduced additional complexity to the interpretation of the findings. Due to the retrospective design, genetic testing for the *PRNP* gene was not possible, and postmortem confirmation was unavailable. Therefore, diagnosis relied on clinical presentation and paraclinical data, including laboratory and neuroimaging findings. Although PSG recordings were scored by an experienced rater in accordance with the AASM criteria, overnight PSG could only be conducted for a single night in all but one patient, which may have introduced a first‐night effect.

## 5 Conclusion

In our population, similar to the literature (Kang et al. 2016b; Landolt et al. 2006), insomnia and daytime sleepiness with disrupted microstructure of sleep were prominent findings. Central or mixed apnea was also remarkable. As a new initial finding, we have seen the Cheyne–Stokes pattern at the early phase of disease in one patient. Clinically and electrophysiologically, REM parasomnia rather than NREM parasomnia was also more frequent in our CJD patients.

All sleep problems might be more common than it is thought in CJD. These may be underestimated or neglected among other symptoms. Especially, initial sleep findings such as central apnea–Cheyne–Stokes pattern can be a disease marker in patients with concomitant mild cognitive impairment in case of an absence of other diagnoses.

Therefore, sleep patterns should be carefully questioned in every patient and caregiver. PSG can be used to clarify the clinical disparities in the future.

## Author Contributions

**Ezgi Demirel**: data curation, formal analysis, writing – original draft, investigation. **Gul Yalcin‐Cakmakli**: formal analysis, investigation, supervision, writing – review and editing. **Ezgi Yetim**: formal analysis, investigation, supervision, writing – review and editing. **Serap Saygi**: conceptualization, formal analysis, methodology, supervision, writing – review and editing. **F. Irsel Tezer**: conceptualization, formal analysis, methodology, supervision, project administration, writing – review and editing.

## Funding

The authors received no specific funding for this work.

## Ethics Statement

This study adhered to the principles outlined in the Declaration of Helsinki. Approval was obtained from the Ethics Committee of Hacettepe University. Informed consent was obtained from all individual participants included in the study.

## Conflicts of Interest

The authors declare no conflicts of interest.

## Data Availability

The data that support the findings of this study are available from the corresponding author, F. Irsel Tezer, upon reasonable request.
